# Clinical presentations and outcomes of celiac disease in children and adolescents at a tertiary care center in Lebanon

**DOI:** 10.3389/fped.2025.1527114

**Published:** 2025-01-22

**Authors:** Dana Andari, Rima Hanna-Wakim, Sarah Khafaja, Nadine Yazbeck

**Affiliations:** ^1^ Global Smile Foundation, Norwood, MA, United States; ^2^ Division of Pediatric Infectious Diseases, Department of Pediatrics and Adolescent Medicine, American University of Beirut, Beirut, Lebanon; ^3^ Department of Pediatrics and Adolescent Medicine, American University of Beirut, Beirut, Lebanon; ^4^ Division of Pediatric Gastroenterology, Department of Pediatrics and Adolescent Medicine, American University of Beirut, Beirut, Lebanon

**Keywords:** celiac disease, gluten-free diet, growth, Mediterranean diet, children

## Abstract

**Introduction:**

Studies on the clinical presentation of celiac disease and its impact on the growth of children in Lebanon are limited. The aim of this 10-year-retrospective study was to describe the common clinical presentations, diagnostic modalities, and the effect of the gluten-free- diet (GFD) on the growth of children and adolescents with celiac disease.

**Methods:**

This was a retrospective chart review of subjects aged 6 months to 18 years who visited the Pediatric Gastroenterology clinic at the American University of Beirut Medical Center (AUBMC) between January 1, 2013, and June 30, 2023, and who were diagnosed with celiac disease based on serological markers and/or changes on histology of the small intestinal mucosal biopsies for those who underwent upper endoscopy, or HLA typing expressing the HLA-DQ2 or DQ8 gene for few subjects.

**Results:**

The study included 90 patients with celiac disease, of whom 64 were newly diagnosed during the study period. The mean age at diagnosis of celiac disease was 6.93 years. Females represented 60% of the pediatric subjects with celiac disease. The most common symptoms reported were abdominal pain (51.1%), weight loss or failure to thrive (45.6%), and diarrhea (24.4%). There was a significant increase in the mean weight-for-age *Z*-score (WAZ) and mean body mass index (BMI)-for-age *Z*-score (BMIZ) 12 months following initiation of GFD; however, the change in height-for-age *Z*-score (HAZ) at 12 months was not statistically significant. Half of the subjects were in remission at the last clinic follow-up.

**Conclusion:**

The most common symptoms that children with celiac disease in this cohort presented with are diarrhea, abdominal pain and failure to thrive. In this cohort, there was a significant increase in the weight parameters with no significant change in the height at 12 months after initiation of the GFD. The recognition of early manifestations, early diagnosis and strict adherence to the diet are of paramount importance to prevent long term complications.

## 1 Introduction

The effect of pediatric gastrointestinal autoimmune diseases including celiac disease on nutrition and growth in children and adolescents can be devastating (1). Growth failure is related to poor appetite, abdominal symptoms, malabsorption, in addition to genetic factors. Celiac disease was found to be the leading cause for short stature, surpassing even growth hormone deficiency (2). There are only few studies assessing the growth trajectory of affected children longitudinally. A study by Kahrs et al. found that children diagnosed with celiac disease tend to be significantly shorter at 12 months of age as compared to controls (3). Another study from the Netherlands showed a decrease in the overall growth trajectory from 6 months to 6 years of age in patients with celiac disease (4).

The gluten-free- diet GFD is currently the only available treatment for patients with celiac disease along with necessary nutritional supplementation such as vitamin D, calcium and iron (5). Following a gluten-free-diet, by avoiding gluten-containing grains, wheat, rye and barley, typically promotes catch-up growth and normalization of weight and leads to clinical remission and normalization of serological markers and histological findings (5–9). In addition, GFD was associated with improvements in serum vitamin D and iron levels as demonstrated in previous studies, and this was attributed to enhanced intestinal absorption (10). However, adherence to the GFD, in the developing world, is challenging mainly in the pediatric and adolescent population due to several barriers, including limited gluten-free options, high costs, poor palatability of the alternatives, insufficient awareness about the importance of the diet, and conflicts between children and their caregivers regarding food choices (5).

There are limited studies on the clinical presentation of celiac disease and its effect on growth of children in the Middle East and North African (MENA) region. This 10-year-retrospective study, aimed to describe the common clinical presentations, diagnostic modalities, and the effect of the gluten-free-diet on the growth of children and adolescents with celiac disease followed at the American University of Beirut Medical Center, a tertiary care center in Lebanon.

## 2 Materials and methods

### 2.1 Study design

We conducted a retrospective chart review of pediatric subjects diagnosed with celiac disease who were followed at the Pediatric Gastroenterology clinic at the American University of Beirut Medical Center (AUBMC) between January 1, 2013, and June 30, 2023.

All subjects were identified retrospectively, and their charts were reviewed through medical records by looking at the following ICD-9 and ICD-10 code for clinic visits for “celiac disease”. The study was approved by the institutional review board (IRB) at AUBMC (IRB approval number: BIO-2023-0174).

### 2.2 Inclusion and exclusion criteria

The subjects included in the study were patients aged 6 months to 18 years diagnosed with celiac disease based on: 1- serological markers including anti-tissue transglutaminase antibody (tTGA) IgG and IgA and anti-endomysial antibody (EMA) IgA (EMA-IgA), and/or; 2- changes on histology of the small intestinal mucosal biopsies with villous atrophy for patients who underwent upper endoscopy, or; 3- HLA typing expressing the HLA-DQ2 or DQ8 gene for patients who refused endoscopy. The EMA is done as a qualitative measure, whereas the tTGA provides quantitative values (for tTGA IgG negative is less than 10 U/ml, and for tTGA IgA negative is less than 4 U/ml). Total blood IgA levels were measured to rule out IgA deficiency. All initial tests were performed while patients were on a gluten-containing diet.

For subjects who did not undergo upper endoscopy and biopsy, the new ESPGHAN guidelines were used for the diagnosis of pediatric celiac disease, where a no-biopsy approach can be followed in patients with tTGA IgA levels ≥10 times the upper limit (11).

Subjects with an unconfirmed diagnosis of celiac disease, those who were lost to follow up and subjects in whom the diagnosis of celiac disease was refuted, were excluded from the study.

### 2.3 Data collection and study variables

Data extracted from medical records included age, gender, clinical presentation, anthropometric measurements, laboratory results such as serological markers, hemoglobin, iron and serum 25-hydroxyvitamin D levels, small intestinal biopsy results, and HLA typing, when available.

The measurements of weight and length/height recorded in the charts of the included subjects were taken by a trained pediatric nurse. The weight of children below the age of two years and below the weight of 20 Kilograms (kg) was measured using a regularly calibrated infant scale with only a clean diaper on. The length of these children was measured in the supine position using a rigid pediatric length measuring board with a range from 0 to 100 cm. Children above the age of two years were weighed barefoot standing on a regularly calibrated scale. Their height was measured standing on a stadiometer with a measuring range of 85–200 cm. The body mass index (BMI) was calculated as the weight in kg divided by height in meters (m) squared.

The anthropometric parameters were expressed as *Z*-scores relative to the National Center for Health Statistics standards using the Baylor College of Medicine calculator [weight-for-age *Z*-score (WAZ), height-for-age *Z*-score (HAZ), and BMI-for-age *Z*-score (BMIZ)] (12).

### 2.4 Definitions

Stunting was defined as HAZ less than 2 standard deviations relative to the normal standards; while wasting or undernutrition was defined as BMIZ less than 2 standard deviations relative to the normal standards. Underweight was defined as WAZ less than 2 standard deviations relative to the normal standards (13, 14).

Remission was defined as symptoms' resolution, and normalization of celiac disease serology after following a GFD. Improved but not in remission was defined as subjects who showed clinical and/or serological improvement but had not yet reached full remission. Relapse was defined as recurrence of symptoms or the reappearance of positive antibody results after clinical and serological normalization.

### 2.5 Statistical analysis

All statistical analysis were performed using the Statistical Package for the Social Sciences program [SPSS, version 23.0 for Windows (IBM, Armonk, NY)]. Simple descriptive statistics were used to describe the subjects' demographics and characteristics, and were represented as frequency and percentages. Continuous variables were reported as mean and range.

The change in growth parameters and hemoglobin, iron and vitamin D values was calculated at 6 and 12 months with the statistical significance determined using the paired *t*-test. A *p*-value less than 0.05 was considered statistically significant.

## 3 Results

**Figure 1:**
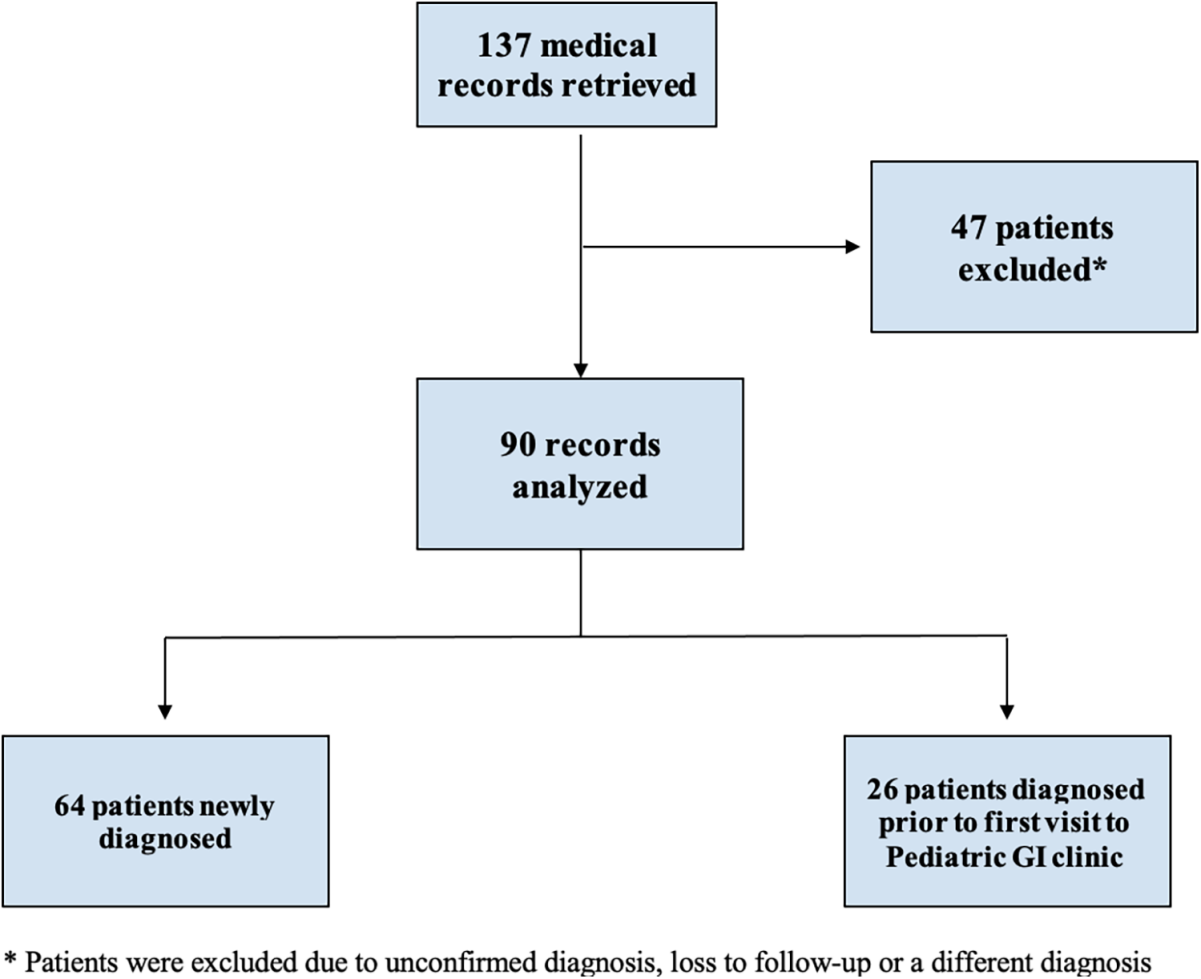
Flowchart of identification and inclusion criteria.

We identified 137 subjects with a diagnosis of “celiac disease”, out of which 47 were excluded due to the following reasons: unconfirmed diagnosis, patient lost to follow-up, or the diagnosis of celiac disease was refuted. Therefore, 90 subjects met the inclusion criteria and were reviewed and included in the statistical analysis. Out of the 90 included subjects, 64 were newly diagnosed and thus included in the analysis of the growth parameters and laboratory value temporal changes. The remaining 26 subjects were diagnosed prior to the first presentation and were already maintained on GFD. These subjects were included in the analysis of the baseline clinical characteristics, pathology results and final outcomes (Figure 1).

### 3.1 Baseline characteristics and clinical presentation

The mean age at first presentation to the Pediatric Gastroenterology Clinic of the 90 subjects was 8.28 years (range 1.1–17.9 years) while the mean age at diagnosis was 6.93 years (range 1.2–16.25 years). The average duration of follow-up was 1.37 years (range 0–9.8 years); therefore, the last follow up results retrieved were taken at 1-year following the initial presentation.

Table 1 represents the demographic and baseline characteristics of the subjects. Among the 90 included subjects, 54 (60%) were females. The majority of the subjects did not have any underlying comorbidities; however, 17.8% had a past medical history of autoimmune diseases such as type 1 diabetes mellitus. In addition, 10% of the subjects had associated gastrointestinal (GI) diseases which included eosinophilic esophagitis or inflammatory bowel disease (IBD). Regarding the family history, 10% of the subjects had a positive family history of autoimmune diseases including type 1 diabetes mellitus or rheumatoid arthritis, and 17.8% of subjects had a family history of GI diseases including colon cancer or IBD. Family history of celiac disease was present in 11.1% of subjects (Table 1).

**Table 1:** Baseline characteristics and clinical presentation of the study population.

	Frequency, *n* (%) *N* = 90
Gender	
Female	54 (60.0)
Past medical history	
Autoimmune diseases^a^	16 (17.8)
Gastrointestinal diseases^b^	9 (10.0)
None	65 (72.2)
Positive family history	
Autoimmune diseases^c^	9 (10.0)
Gastrointestinal diseases^d^	16 (17.8)
Celiac disease	10 (11.1)
No	65 (72.2)
Clinical Presentation	
Abdominal Pain	46 (51.1)
Diarrhea	22 (24.4)
Constipation	11 (12.2)
Weight loss Failure to thrive	41 (45.6)
Extraintestinal symptoms^e^	14 (15.6)
Loss of Appetite	13 (14.4)
Recurrent oral ulcers	4 (4.4)
Fatigue	13 (14.4)
Other clinical presentation^f^	12 (13.3)
Needed hospital admission at presentation	11 (12.2)

^a^ Autoimmune diseases such as type 1 diabetes mellitus.

^b^ Gastrointestinal diseases included eosinophilic esophagitis or inflammatory bowel disease (IBD).

^c^ Autoimmune diseases such as type 1 diabetes mellitus or rheumatoid arthritis.

^d^ Gastrointestinal diseases include colon cancer, Inflammatory Bowel Disease (IBD) or celiac disease.

^e^ Extraintestinal symptoms: joint pain or skin rash.

^f^ Other clinical presentation: Abdominal distension, recurrent infections, hematochezia, type 1 diabetes mellitus.

At the first development of symptoms, subjects often presented with more than one symptom. The most common clinical symptom reported was abdominal pain in 46 subjects (51.1%), followed by weight loss or failure to thrive in 41 (45.6%) subjects, and diarrhea in 22 (24.4%) subjects. There were 12 (13.3%) subjects who had other disorders that warranted screening for celiac disease. These included prior diagnosis of type 1 diabetes mellitus, abdominal distention, recurrent infections, or hematochezia. Out of the 90 subjects, 12.2% required a hospital visit and/or admission due to abdominal pain and diarrhea prior to presentation (Table 1).

### 3.2 Anthropometric parameters

**Figure 2:**
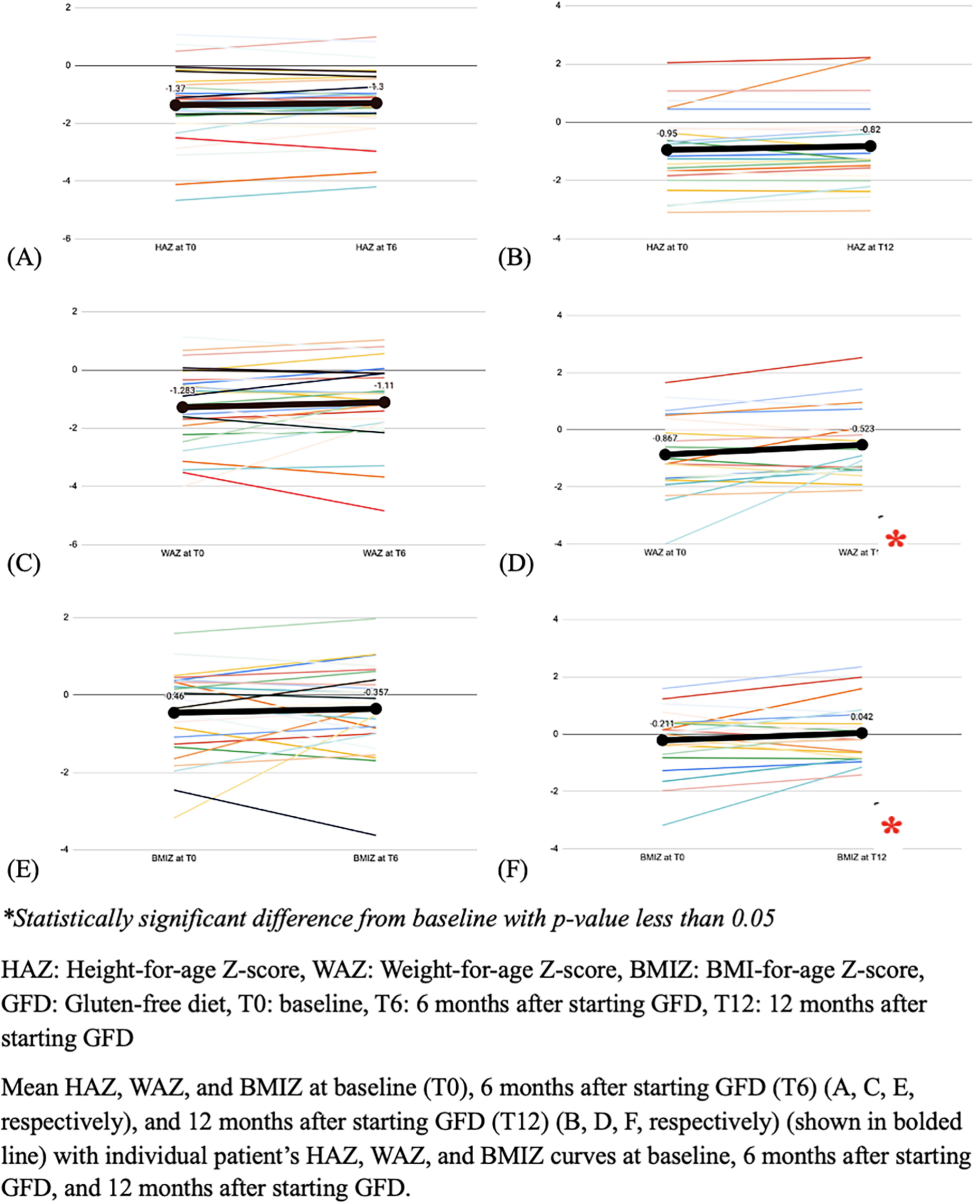
The mean height, weight and BMI Z-scores, at baseline and follow-up (*N* = 64).

Anthropometric indices during follow up are detailed in Figure 2. At the time of first presentation, stunting was identified in 9 (14.1%) subjects, underweight in 11 (17.2%) subjects, and wasting in 4 (6.5%) subjects, with no significant change in these percentages, at 6 months and 12 months after the start of the GFD (*p*-value > 0.05).

There was no significant change of the WAZ 6 months after the start of the GFD, while there was a significant increase of 0.344 in the mean WAZ at 12 months following the start of the GFD (*p*-value = 0.022). Similarly, there was no significant change of BMIZ at 6 months, but there was a significant increase of 0.253 at 12 months (*p*-value = 0.047). In contrast, there was no significant change in the HAZ at either 6- and 12-months follow-up (*p*-value 0.0721 and 0.085, respectively) (Figure 2).

### 3.3 Laboratory evaluation

At baseline, the mean hemoglobin level was 12.58 ± 1.34 g/L, with 36.5% of subjects having a hemoglobin level less than 12 g/L. The mean serum iron level was 63.10 ± 44.70 mcg/dl (normal: 37–160 mcg/dl), and the mean serum vitamin D level was 26.32 ± 15.53 ng/ml (adequate: > 20 ng/ml).

Among the 48 subjects tested for EMA-IgA at baseline, 44 subjects (91.7%) had positive serology. At 6 and 12 months after starting GFD, 11 out of 16 subjects (68.8%) and 12 out of 19 subjects (63.2%), respectively, continued to test positive for EMA-IgA.

At presentation, 70.5% of the 61 subjects who were tested for tTGA-IgA had levels greater than 100 U/ml. This percentage decreased to 16% and 4.6%, at 6 and 12 months, respectively, following the initiation of GFD (Table 2).

**Table 2:** Laboratory values and serology markers at baseline (T0), 6 months after starting the GFD (T6) and 12 months after starting the GFD (T12).

Laboratory values	T0, mean	T6, mean	T12, mean
Hemoglobin (g/L); normal: ≥ 12	*N* = 41 12.58	*N* = 23 12.97	*N* = 19 12.81
Iron (mcg/dl); normal: 37–160	*N* = 30 63.10	*N* = 19 80.46	*N* = 14 66.39
Vitamin D (ng/ml); normal: > 20	*N* = 27 26.32	*N* = 20 24.13	*N* = 18 23.45
Serology Markers	T0, *n* (%)	T6, *n* (%)	T12, *n* (%)
Endomysial IgA	*N* = 48	*N* = 16	*N* = 19
Positive	44 (91.7)	11 (68.8)	12 (63.2)
Negative	4 (8.3)	5 (31.2)	7 (36.8)
Anti-transglutaminase IgA (U/ml)	*N* = 61	*N* = 25	*N* = 22
<4	6 (9.8)	9 (36.0)	14 (63.6)
4–100	12 (19.7)	12 (48.0)	7 (31.8)
>100	43 (70.5)	4 (16.0)	1 (4.6)
Anti-transglutaminase IgG (U/ml)	*N* = 31	*N* = 20	*N* = 17
<10	13 (41.9)	14 (70.0)	13 (76.5)
10–100	14 (45.2)	6 (30.0)	4 (23.5)
>100	4 (12.9)	0 (0.0)	0 (0.0)

To note that the EMA is done by indirect immunofluorescence assay (IFA) by Euroimmun as a qualitative measure, whereas the tTGA provides quantitative values (for tTGA IgG negative is less than 10 U/ml, and for tTGA IgA negative is less than 4 U/ml). tTGA IgG and IgA are an enzyme-linked immunosorbent assay (ELISA) based; automated, *in vitro* test performed on Alegria® from Orgentec (ORG 240A-24, ORG 240G-24).

Moreover, eight subjects had HLA typing (specifically HLA DQ2 and DQ8) performed showing positive results.

### 3.4 Pathology

Out of the 90 subjects with celiac disease, 51 subjects underwent upper gastrointestinal endoscopy with duodenal biopsies. The remaining 39 subjects did not undergo the procedure due to lack of financial coverage, fear of endoscopy and sedation, or loss to follow up. Among the 51 subjects who had biopsies, 49 (96%) subjects showed villous atrophy on pathology results. Of those with villous atrophy, 49.0% had partial villous atrophy, 30.6% had subtotal villous atrophy, and 20.4% had total villous atrophy.

### 3.5 Treatment and outcomes

All subjects had consultation with a certified pediatric dietitian who thoroughly discussed the GFD with both the subjects and caregivers and provided individualized diet plans taking into consideration the Mediterranean diet followed in Lebanon.

**Figure 3:**
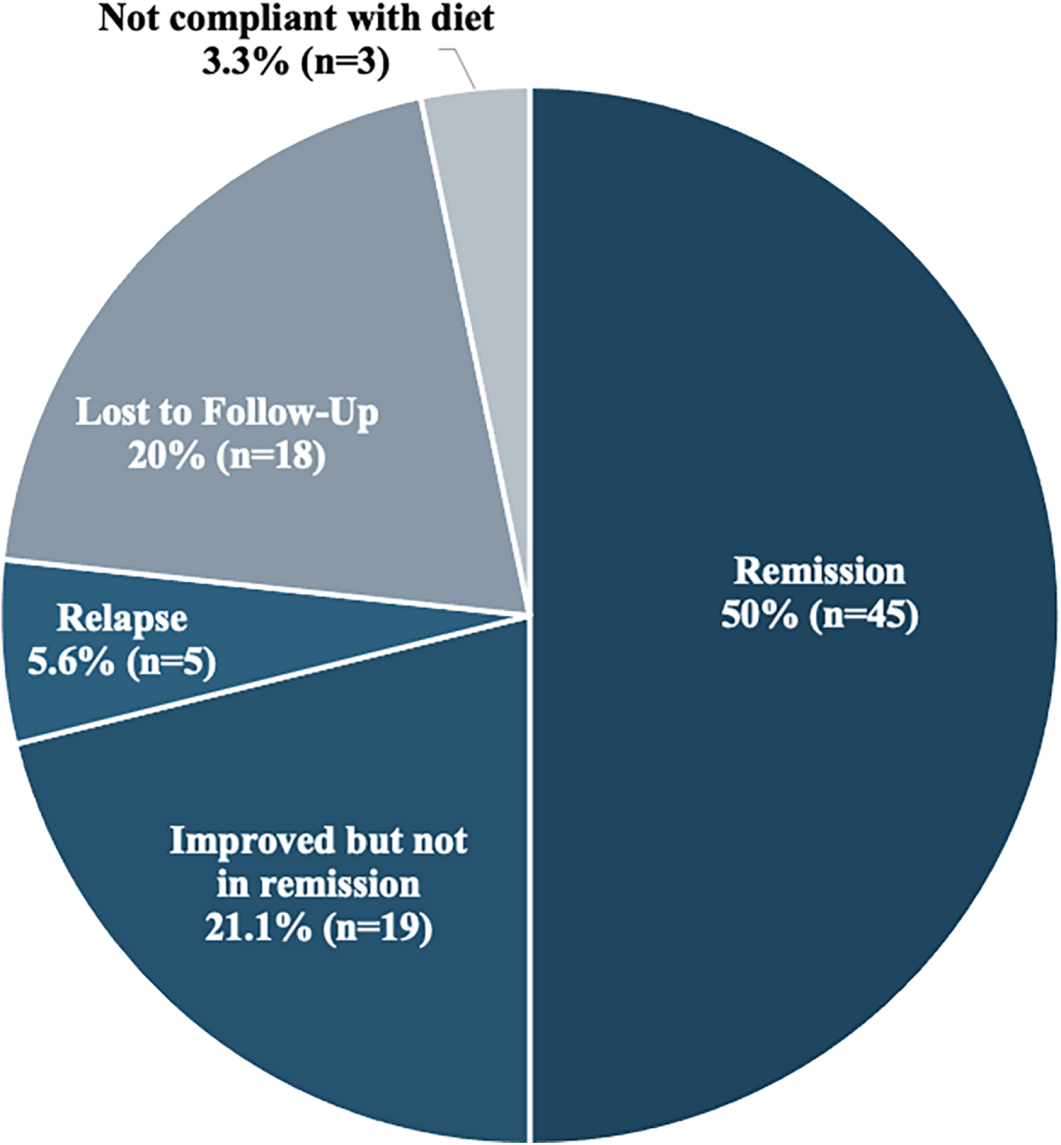
Outcomes at last follow-up (*N* = 90).

At their last follow up visit (range 0–9.8 years), half of the subjects were in remission while 19 (21.1%) subjects had an improvement in their symptoms and serological values. Relapse, defined as re-development of symptoms or positive antibody results after clinical and serological normalization, occurred in 5 (5.6%) subjects relapsed. Three subjects were not adherent to a strict GFD which resulted in no changes in their clinical symptoms and/or serological tests. Loss to follow up was documented in 20% of subjects (Figure 3).

## 4 Discussion

In this cohort, there was a greater percentage of females (60%) as compared to males that were diagnosed with celiac disease, which concurs with the evidence that celiac disease is twice as frequent in females than males (15). The mean age at diagnosis of celiac disease was 8.28 years in our cohort which is similar to studies from Oman and Finland in which the mean age at diagnosis was 7 years and 7.2 years respectively (16, 17). In our group, the mean years of follow-up was 1.37 years with a range of 0–9.8 years. We postulate that there are several potential factors for the lack of follow up in our setting including the COVID-19 pandemic, the economic crisis, and the armed conflicts in Lebanon.

The most common symptoms reported by these 90 subjects were abdominal pain (51.1%), weight loss or failure to thrive (42.2%), and diarrhea (23.3%). Previous studies linked the symptoms of celiac disease to growth failure due to abdominal pain and malabsorption (2). A study done in Morocco showed that the main consultation reason was diarrhea (46%) and growth delay (32.4%) (18). To note that in our cohort, the majority of the subjects were symptomatic at the time of first presentation which is unlike other studies where children are asymptomatic at the time of diagnosis (19). This may be due to limited resources and specialists in the field leading to overlooked diagnoses until a later stage.

Screening for celiac disease usually starts with serological testing with EMA IgA and IgG, tTGA IgA and IgG, antigliadin antibodies IgA and IgG, and a new generation of antigliadin antibodies to deamidated synthetic gliadin peptides (20). These tests have a very high sensitivity and specificity for the disease (21–23). In our center, the most common tests used are the EMA-IgA and tTGA IgA and IgG. Previously, the gold standard for the diagnosis of celiac disease was known to be small intestinal biopsy (21, 22, 24, 25). However, in line with the new ESPGHAN guidelines for the diagnosis of pediatric celiac disease, a no-biopsy approach can be followed given that the tTGA IgA levels are ≥10 times the upper limit, and there is a shared decision with the caregivers and/or patients (11). Of the 90 subjects in the cohort, only 51 had an endoscopy done, and 49 of those showed villous atrophy on pathology. From the 39 subjects who did not undergo upper endoscopy and duodenal biopsies, 8 subjects had HLA typing. For the rest of the subjects, the diagnosis was established based on the clinical manifestations and the serologic tests. Gidrewicz et al. studied the duration to normalization of serologies and found that approximately 75% of patients with celiac disease strictly adherent to GFD still had abnormal serologies one year later (23). This is similar to our cohort with 63.2% of patients still testing positive for EMA-IgA.

At the time of diagnosis, 14.1% of subjects were stunted, 17.2% were underweight, and 6.5% were wasted. To note that the percentage of children in Lebanon under the age of 5 who were stunted in 2020 was 10.4%, and in the region was 26.2% (26). A study done by Aggarwal et al. showed that patients with celiac disease in India do not necessarily have short stature (27). However, a study done in Pakistan showed that 38% were severely undernourished and 8% were severely stunted with 79% having short stature (28). In a study done in Saudi Arabia, 85% of the patients with celiac disease were stunted with the mean HAZ and the mean WAZ at diagnosis being −3, −2.8 respectively (29). Our cohort had less underweight and wasted patients which may be due to the Mediterranean diet followed in Lebanon.

The prevalence of iron deficiency anemia (IDA) ranges from 2.3 to 33% in children with celiac disease (10). In our cohort, 26.7% of subjects who had iron studies at baseline were iron deficient. Furthermore, the mean serum vitamin D level in the cohort was in the lower range of desirable. This is in contrast to the literature showing that patients with untreated celiac disease tend to have lower serum vitamin D levels as compared to healthy controls without celiac disease (30). Our cohort's value could be attributed to the low number of subjects who had serum vitamin D levels tested (*n* = 27).

There was a significant increase in the mean WAZ and mean BMIZ (*p*-value = 0.022 and 0.047), and no significant change in the mean HAZ at 12 months after initiation of GFD. A controlled study done in Qatar comparing the effect of the GFD on linear growth in patients with celiac disease showed no significant change in the mean HAZ at the 12-month follow-up. However, the children in the controlled study's cohort were still increasing in height, but at a slower pace (31). It was shown that at 6–12 months after starting the GFD, children with celiac disease tend to achieve a near-normal growth curve for weight, and at 2–3 years after starting the GFD, they tend to achieve a normal growth curve for height (32, 33). This may explain the results in our cohort that showed a significant improvement in WAZ, but not in HAZ indicating that height catch-up might need longer than one year after GFD to occur. Furthermore, a systematic review and meta-analysis done by Xin et al. showed that the GFD has a significant and beneficial effect on weight of patients with celiac disease (34). Another retrospective study comparing patients with celiac disease in Italy and the United States of America (USA) showed that the catch-up growth in children in Italy was slower as compared to those in the USA (35). This was linked to the differences in the lifestyle and culture, which could be applied to the Lebanese population as well since there are limited gluten-free resources and the Mediterranean diet includes several foods containing gluten (35). To date, there are no studies that evaluated the effect of the gluten-free Mediterranean diet on growth in children, which could be an area of exploration in future studies.

At the time of the last follow-up visit, 50% of the subjects were in remission, whereas 21.1% were improving clinically and serologically but were still not in remission. The reason for poor compliance is not completely clear, but lack of awareness about celiac disease manifestations, lack of GFD knowledge or even lack of benefit from the diet have been described as contributing factors (36). Lebanon has limited gluten-free options available and accessible. The Mediterranean diet is rich in grain and wheat which contain gluten making it challenging to follow a strict GFD. In addition, the mean age at diagnosis is between 7 and 8 years of age, which may be a difficult age to convince the child to follow a strict diet. In the pediatric population, compliance to the GFD is 52–95 percent with very minimal adherence to permanent GFD as shown in a study done in North India (9).

### 4.1 Limitations

Although our study is the first to describe the clinical presentations and outcomes of celiac disease in children and adolescents in Lebanon; it is important to note certain limitations. The retrospective design limited our capacity to gather accurate and complete data and did not allow us to have appropriate long-term follow up to evaluate compliance with the GFD. In addition, the study included a small sample size from one tertiary care center in Lebanon which may affect its generalizability and lead to referral bias.

## 5 Conclusion

The most common symptoms that children with celiac disease in this cohort presented with are diarrhea, abdominal pain and failure to thrive. In a resource-constrained country like Lebanon, the diagnosis and management remain challenging in view of the high cost of procedures and the scarcity of affordable gluten-free products. Therefore, opting for more cost-friendly diagnostic parameters such as celiac serologies in specific cases instead of endoscopy may be a feasible alternative as shown in this study and supported by the ESPHGAN guidelines. Celiac disease in children affects negatively growth and development which are expected to improve after initiation of GFD. In this cohort, there was a significant increase in the WAZ and BMIZ and no significant change of HAZ at 12 months after initiation of the GFD. The recognition of early manifestations, early diagnosis and strict adherence to the diet are of paramount importance to prevent long term complications.

Since the Lebanese population follows the Mediterranean diet, we believe that our paper will add to the current literature available and hopefully open discussions for further research on the topic of linking different types of diets to the outcome of patients with celiac disease.

## Data Availability

The raw data supporting the conclusions of this article will be made available by the authors, without undue reservation.

## References

[1] Tsiountsioura M Wong JE Upton J McIntyre K Dimakou D Buchanan E Detailed assessment of nutritional status and eating patterns in children with gastrointestinal diseases attending an outpatients clinic and contemporary healthy controls. Eur J Clin Nutr. (2014) 68(6):700–6. 10.1038/ejcn.2013.286 24424079

[2] Troncone R Kosova R. Short stature and catch-up growth in celiac disease. J Pediatr Gastroenterol Nutr. (2010) 51(Suppl 3):S137–8. 10.1097/MPG.0b013e3181f1dd66 21088537

[3] Kahrs CR Magnus MC Stigum H Lundin KEA Stordal K. Early growth in children with coeliac disease: a cohort study. Arch Dis Child. (2017) 102(11):1037–43. 10.1136/archdischild-2016-312304 28611068

[4] Jansen MA Kiefte-de Jong JC Gaillard R Escher JC Hofman A Jaddoe VW Growth trajectories and bone mineral density in anti-tissue transglutaminase antibody-positive children: the generation R study. Clin Gastroenterol Hepatol. (2015) 13(5):913–20 e5. 10.1016/j.cgh.2014.09.032 25245626

[5] Barada K Abu Daya H Rostami K Catassi C. Celiac disease in the developing world. Gastrointest Endosc Clin N Am. (2012) 22(4):773–96. 10.1016/j.giec.2012.07.002 23083993

[6] Meazza C Pagani S Laarej K Cantoni F Civallero P Boncimino A Short stature in children with coeliac disease. Pediatr Endocrinol Rev. (2009) 6(4):457–63. 19550380

[7] Patwari AK Kapur G Satyanarayana L Anand VK Jain A Gangil A Catch-up growth in children with late-diagnosed coeliac disease. Br J Nutr. (2005) 94(3):437–42. 10.1079/BJN20051479 16176616

[8] Saadah OI Zacharin M O'Callaghan A Oliver MR Catto-Smith AG. Effect of gluten-free diet and adherence on growth and diabetic control in diabetics with coeliac disease. Arch Dis Child. (2004) 89(9):871–6. 10.1136/adc.2002.012799 15321869 PMC1763216

[9] Hota D Bhalla K Nanda S Gupta A Mehra S. Beneficial effects of gluten free diet on IgA tissue transglutaminase levels and various growth parameters in celiac disease patients. J Family Med Prim Care. (2019) 8(3):823–7. 10.4103/jfmpc.jfmpc_56_19 31041208 PMC6482799

[10] Talarico V Giancotti L Mazza GA Miniero R Bertini M. Iron deficiency Anemia in celiac disease. Nutrients. (2021) 13(5):1695. 10.3390/nu13051695 34067622 PMC8156426

[11] Husby S Koletzko S Korponay-Szabo I Kurppa K Mearin ML Ribes-Koninckx C European Society Paediatric Gastroenterology, hepatology and nutrition guidelines for diagnosing coeliac disease 2020. J Pediatr Gastroenterol Nutr. (2020) 70(1):141–56. 10.1097/MPG.0000000000002497 31568151

[12] Age-based Pediatric Growth Reference Charts. BMI Z-Score and Percentile Calculator.. https://www.bcm.edu/bodycomplab/BMIapp/BMI-calculator-kids.html.

[13] Sinha RK Dua R Bijalwan V Rohatgi S Kumar P. Determinants of stunting, wasting, and underweight in five high-burden pockets of four Indian states. Indian J Community Med. (2018) 43(4):279–83. 10.4103/ijcm.IJCM_151_18 30662180 PMC6319291

[14] Fact sheets - malnutrition. World Health Organization. (2024). Available online at: https://www.who.int/news-room/fact-sheets/detail/malnutrition#:∼:text=undernutrition%2C%20which%20includes%20wasting%20(low,minerals)%20or%20micronutrient%20excess%3B%20and (accessed June 11, 2024).

[15] Petronzelli F Bonamico M Ferrante P Grillo R Mora B Mariani P Genetic contribution of the HLA region to the familial clustering of coeliac disease. Ann Hum Genet. (1997) 61(Pt 4):307–17. 10.1046/j.1469-1809.1997.6140307.x 9365784

[16] Al-Lawati TT Al-Musawi HS. Celiac disease in Oman: a tertiary centre experience. Oman Med J. (2013) 28(1):70–2. 10.5001/omj.2013.17 23386952 PMC3562981

[17] Savilahti E Kolho KL Westerholm-Ormio M Verkasalo M. Clinics of coeliac disease in children in the 2000s. Acta Paediatr. (2010) 99(7):1026–30. 10.1111/j.1651-2227.2010.01740.x 20199495

[18] Mouslih A El Rhazi K Bahra N Lakhdar Idrissi M Hida M. Celiac disease in Moroccan children: diagnostic characteristics and determinants of diagnosis delay. Cureus. (2023) 15(12):e50800. 10.7759/cureus.50800 38125690 PMC10731523

[19] Nenna R Tiberti C Petrarca L Lucantoni F Mennini M Luparia RP The celiac iceberg: characterization of the disease in primary schoolchildren. J Pediatr Gastroenterol Nutr. (2013) 56(4):416–21. 10.1097/MPG.0b013e31827b7f64 23149808

[20] Volta U Granito A Fiorini E Parisi C Piscaglia M Pappas G Usefulness of antibodies to deamidated gliadin peptides in celiac disease diagnosis and follow-up. Dig Dis Sci. (2008) 53(6):1582–8. 10.1007/s10620-007-0058-0 17985240

[21] Rostom A Murray JA Kagnoff MF. American Gastroenterological association (AGA) institute technical review on the diagnosis and management of celiac disease. Gastroenterology. (2006) 131(6):1981–2002. 10.1053/j.gastro.2006.10.004 17087937

[22] Rostom A Dube C Cranney A Saloojee N Sy R Garritty C The diagnostic accuracy of serologic tests for celiac disease: a systematic review. Gastroenterology. (2005) 128(4 Suppl 1):S38–46. 10.1053/j.gastro.2005.02.028 15825125

[23] Gidrewicz D Trevenen CL Lyon M Butzner JD. Normalization time of celiac serology in children on a gluten-free diet. J Pediatr Gastroenterol Nutr. (2017) 64(3):362–7. 10.1097/MPG.0000000000001270 28231071

[24] Stern M. Working group on serologic screening for celiac D. Comparative evaluation of serologic tests for celiac disease: a European initiative toward standardization. J Pediatr Gastroenterol Nutr. (2000) 31(5):513–9. 10.1097/00005176-200011000-00012 11144436

[25] Lee SK Green PH. Endoscopy in celiac disease. Curr Opin Gastroenterol. (2005) 21(5):589–94. 10.1097/01.mog.0000174218.00333.19 16093775

[26] The work of WHO in the Eastern Mediterranean Region-Annual report of the regional director 2019 2019. Available online at: https://applications.emro.who.int/docs/9789290223467-eng.pdf?ua=1 (Accessed November 01, 2024).

[27] Aggarwal N Dwarakanathan V Singh A Agarwal A Khuttan A Ahmed A Spectrum of height in patients with celiac disease. Indian J Gastroenterol. (2021) 40(6):604–12. 10.1007/s12664-021-01173-9 34921660

[28] Jabeen S Khan AU Ahmed W Ahmad MU Jafri SA Bacha U Disease specific symptoms indices in patients with celiac disease-A hardly recognised entity. Front Nutr. (2022) 9:944449. 10.3389/fnut.2022.944449 36159486 PMC9494589

[29] Saadah OI. The impact of a gluten-free diet on the growth pattern of Saudi children with coeliac disease. J Pak Med Assoc. (2021) 71(5):1388–93. 10.47391/JPMA.1308 34091620

[30] Lu C Zhou W He X Zhou X Yu C. Vitamin D status and vitamin D receptor genotypes in celiac disease: a meta-analysis. Crit Rev Food Sci Nutr. (2021) 61(12):2098–106. 10.1080/10408398.2020.1772716 32508121

[31] Soliman AT Laham M Jour C Shaat M Souikey F Itani M Linear growth of children with celiac disease after the first two years on gluten- free diet: a controlled study. Acta Biomed. (2019) 90(8-S):20–7. 10.23750/abm.v90i8-S.8515 31544803 PMC7233684

[32] Damen GM Boersma B Wit JM Heymans HS. Catch-up growth in 60 children with celiac disease. J Pediatr Gastroenterol Nutr. (1994) 19(4):394–400. 10.1097/00005176-199411000-00005 7876992

[33] Barr DG Shmerling DH Prader A. Catch-up growth in malnutrition, studied in celiac disease after institution of gluten-free diet. Pediatr Res. (1972) 6(5):521–7. 10.1203/00006450-197205000-00006 4340240

[34] Xin C Imanifard R Jarahzadeh M Rohani P Velu P Sohouli MH. Impact of gluten-free diet on anthropometric indicators in individuals with and without celiac disease: a systematic review and meta-analysis. Clin Ther. (2023) 45(12):e243–e51. 10.1016/j.clinthera.2023.09.018 37903705

[35] Sansotta N Guandalini S Romano S Amirikian K Cipolli M Tridello G The gluten free diet’s impact on growth in children with celiac disease in two different countries. Nutrients. (2020) 12(6):1547. 10.3390/nu12061547 32466557 PMC7352316

[36] Rostami K Malekzadeh R Shahbazkhani B Akbari MR Catassi C. Coeliac disease in Middle Eastern countries: a challenge for the evolutionary history of this complex disorder? Dig Liver Dis. (2004) 36(10):694–7. 10.1016/j.dld.2004.05.010 15506671

